# Tutorless board game as an alternative to tabletop exercise for disaster response training: perception of interaction engagement and behavioral intention

**DOI:** 10.1186/s12909-023-04356-4

**Published:** 2023-06-12

**Authors:** Keng Sheng Chew, Shirly Siew-Ling Wong, Izzah Safiah binti Tarazi, Janet Weilly Koh, Nor Azeriyatul ‘Ain binti Ridzuan, Syed Azrai Shah bin Wan Allam

**Affiliations:** 1 Faculty of Medicine and Health Sciences, Universiti Malaysia Sarawak, 94300 Kota Samarahan, Sarawak, Malaysia; 2 Faculty of Economics and Business, Universiti Malaysia Sarawak, 94300 Kota Samarahan, Sarawak, Malaysia

**Keywords:** Board game, Disaster training, Gamification, Interaction engagement, Behavioral intention

## Abstract

**Background:**

Although tabletop exercise is a commonly used method for disaster response training, it is labor-intensive, requires a tutor for facilitation and may not be ideal in a pandemic situation. Board game is a low-cost and portable alternative that can be utilized for this purpose. The purpose of this study was to compare the perception of interaction engagement and behavioral intention to use a newly developed board game with tabletop exercise for disaster training.

**Methods:**

Using the Mechanics-Dynamics-Aesthetics’ (MDA) framework, a new, tutorless educational board game known as the Simulated Disaster Management And Response Triage training (“SMARTriage”) was first developed for disaster response training. Subsequently, the perceptions of 113 final year medical students on the “SMARTriage” board game was compared with that of tabletop exercise using a crossover design.

**Results:**

Using Wilcoxon signed rank test, it was that found that tabletop exercise was generally rated significantly higher (with *p* < 0.05) in terms of perceived usefulness, perceived ease of use and behavioral intention compared to tutorless “SMARTriage” board game. However, in terms of attitude and interaction engagement, there was no significant difference between these two learning methods for most of the items.

**Conclusion:**

Although a clear preference for tutorless board game was not demonstrated, this study suggests that board game was not inferior to tabletop exercise in fostering interaction engagement suggesting that “SMARTriage” board game could potentially be used as an adjunct for teaching and learning activities.

**Supplementary Information:**

The online version contains supplementary material available at 10.1186/s12909-023-04356-4.

## Introduction

Disaster response management is an important subject that should be incorporated in medical and nursing education [1, 2]. This subject should be taught using a multi-modal approach by leveraging on a variety of educational tools [1]. One of these frequently used tools is tabletop exercise [1, 3–5]. Tabletop exercise is a cost-efficient, classroom-type disaster response simulation activity where students are presented with an unfolding scenario; and through the iterative interactions with one another, the students learn to make decisions in a disaster scenario [5]. Tabletop exercise, however, is labor-intensive and it requires a tutor to facilitate or coordinate the session. Only a limited number of case scenario combinations could be discussed for the entire class in a tabletop exercise. As different students are assigned different roles and tasks, the learning experience of each student cannot be standardized [5]. Furthermore, tabletop exercises would require the gathering of the entire group of students which may not be ideal if the size of the group is becoming too large particularly in a pandemic situation.

One option that could be used as an alternative or a supplement to tabletop exercise in disaster training is through games and gamification strategies. Game is defined as a set of problem-solving activities approached with a playful attitude [6] whereas gamification is defined as the application of game design elements in a non-game context [7]. Generally, a major advantage of using games and gamification strategies for teaching and learning is the fact that it affords the opportunity for students to explore and to learn from mistakes in a safe environment [8]. A type of low-cost game that is portable enough and does not require internet connection or any electronic device to play is the board game.

To the best of our knowledge, although a number of games for disaster response training had been described in literature [9], the use of board game in disaster response training specifically designed for healthcare students is rarely reported. One recently published board game for medical students’ disaster training was the “AFTERSHOCK” game designed by a group of German medical students. The aim of this board game is to train medical students to respond to the aftermath of an earthquake [10]. The authors found that “AFTERSHOCK” game was a suitable and acceptable method for disaster training. In that study, however, the level of interaction engagement was not measured and neither was a comparison between the board game with another conventional teaching and learning method made.

To fill in this gap, we conducted a study on a newly developed disaster training board game with the primary objective of comparing the perception of interaction engagement and behavioral intention to use this board game as compared to the conventional tabletop exercise.

## Methodology

### Setting

In our institution, i.e., Universiti Malaysia Sarawak (UNIMAS), disaster response training is part of our final year medical students’ emergency medicine rotation. In this training, a 1 to 2-h tutorial session was first given to all students on the basic concepts of disaster, the different phases of a disaster, the principles of multi-agency field and hospital management and the contingency planning to minimize the impact of any future recurrence. This was followed by a practical session on disaster triaging and response skills, conventionally conducted using tabletop exercise.

### Study design

We divided our study into two main phases. In Phase 1, we first described the processes of designing a new educational board game known as the Simulated Disaster Management And Response Triage training (“SMARTriage”) (Phase 1 of our project). Subsequently, in Phase 2, we compared the medical students’ perception on this tutorless “SMARTriage” board game with the conventional tabletop exercise.

The board game was designed in the year 2020 and the study was conducted throughout in the entire year 2021 (2 semesters). The board game sessions were conducted in our clinical skills training room. The room was spacious enough to accommodate about 12—14 students performing cardiopulmonary resuscitation on manikins at any one time. The tabletop exercise sessions were conducted in smaller tutorial rooms that allow students to break out into 3 smaller groups. Ethical approval was obtained from the institutional medical research ethics board of UNIMAS (reference no UNIMAS/NC-21.02/03–02 Jld.4 (22)).

### Participants

For Phase 1, all authors participated in group discussions with the aim of designing and developing the board game. For Phase 2, the entire cohort of 113 final year medical students were recruited sequentially in groups of 43, 36 and 34 students. The medical students had to be recruited sequentially as they came in groups of 30 – 40 students per group for their emergency medicine rotation.

### Materials

For Phase 1, the Mechanics-Dynamics-Aesthetics’ (MDA) framework by Hunicke et al. [11] for game design was applied. “Mechanics” refers to a set of rules that define what can or cannot be done in the game. “Dynamics” refers to the players’ experience and interaction with the rules of the game. “Aesthetics” refer to the intended emotional experience evoked when a player participates in the game. Hunicke et al. [11] further described a taxonomy of at least 9 types of aesthetics, i.e., sensation (game as sense-pleasure), fantasy (game as make-believe), narrative (game as drama), challenge (game as obstacle course), fellowship (game as social framework), discovery (game as uncharted territory), expression (game as self-discovery) and submission (game as pastime). In “SMARTriage” board game, the intended aesthetics are (1) fellowship and (2) challenge (i.e., the experience of different players collaborating, rather than competing, to beat the time limit in order to correctly triage and transfer all patients to the definitive wards). We further considered the dynamics of our board game from five different elements of user experience described by von Wangenheim et al. [12]. These five elements are (1) the immersion element; (2) the challenge element; (3) the competence element; (4) the fun element and (5) the social interactions element.

Phase 2 of the study was conducted using the psychometric instrument that we adapted from a previously validated instrument [13]. However, we differed from the previous instrument in that we included “behavioral intention” as our dependent variable. Furthermore, in the previous instrument, students’ engagement was further divided into two separate constructs: (1) “skill engagement” (referring to learning skills such as taking good notes and listening well in class, etc.) and (2) “interaction engagement” [13]. In our study, “skill engagement” was excluded in our instrument as these items (e.g., measuring “taking good notes in classroom”, “listening carefully in classroom”, etc.) were deemed irrelevant in our practical disaster response training. All items in “interaction engagement” construct listed in the previous instrument were included in our instrument development except for the item “asking questions when I did not understand”. This was because unlike the tabletop exercise, the “SMARTriage” board game session was purported to be a tutorless session (except at the beginning of the session for briefing on how to play and the end of the session for debriefing). Hence, it was felt that it would not be a fair comparison to include this item “asking questions when I did not understand” as the amount of contact time with the tutor was significantly low in the “SMARTriage” compared to in tabletop exercise.

### Procedures

For Phase 1, group discussions were held during the first 2 months of this study to develop the mechanics, dynamics and aesthetics components of “SMARTriage” board game using the framework by Hunicke et al. [11]. In the first two sessions, all authors KSC, SSLW, IST, JWK, NAAR and SASWA attended and contributed to this development and design. Author KSC (who is an emergency physician with special interest in medical education) then invited three emergency residents (Dr. ET, Dr. YYT and Dr. CYT) from the emergency and trauma department of Sarawak General Hospital (who were not part of this project) to contribute some case scenarios for this game. The subsequent iterative process of vetting and fine tuning of these case scenarios were conducted through emails and the short messaging application, WhatsApp. The details of the conduct of both tabletop exercise and board game are detailed in Table 1.

**Table 1:** Comparison of the learning objectives, combination of case scenarios and conduct of tabletop exercise and “SMARTriage” board game

Tabletop exercise	“SMARTriage” board game
Learning objectives: At the end of this session, the students are able to: 1. perform pre-hospital triage using SMART system in a disaster 2. perform intra-facility and inter-facility transfer for disaster victims taking into consideration the provisional diagnoses generated	Learning objectives: At the end of this session, the students are able to: 1. perform pre-hospital triage using SMART system in a disaster 2. perform intra-facility and inter-facility transfer for disaster victims taking into consideration the provisional diagnoses generated
Scenario: At 3 am, the emergency department of “Hospital A” (a tertiary hospital) received call from a passerby saying that a motor-vehicle accident at km 5 of a highway involving 3 vehicles had just occurred	Scenario: At 3 am, the emergency department of “Hospital A” (a tertiary hospital) received call from a passerby saying that a motor-vehicle accident at km 5 of a highway involving 3 vehicles had just occurred
Number of clinical cases: 6—7 cases Example of case: *“Patient B” (Driver of car) is a 40-year-old man. Vital signs: respiratory rate 40/min, regular; pulse rate 110 bpm, good volume* *Drowsy but responds to pain stimuli. Scalp laceration wound at left temporal region and swelling over left forearm.”*	Number of cases: 8 cases in the “Disaster zone”, 4 cases in the “Tertiary Hospital” and 4 cases in the “District Hospital” Example of case: *“A 27-year old – respiratory rate 45/min – complaining of difficulty in breathing – decreased chest movement noted on left side”*
Combination of cases: Fixed combination	Combination of cases: As there are 50 “Disaster zone” cards, the number of combination of cases in a game with 8 cards are = 50 × 49 × 48 × 47 × 46 × 45 × 44 × 43 = 21 × 10^12^ As there are 9 “Tertiary Hospital” cards, the number of combination of cases in a game with 8 cards are = 9 × 8 × 7 × 6 = 1728 As there are 9 “District Hospital” cards, the number of combination of cases in a game with 8 cards are = 9 × 8 × 7 × 6 = 1728
Presence of tutor/facilitator: Throughout the entire session	Presence of tutor/facilitator: Only at the beginning of the session to brief the students on how to play the game and toward the end for debriefing
**Instruction:** The class is divided into 3 groups: Group 1 plays the role of the staff in the emergency department/medical base of Hospital A (the major tertiary hospital where all referral cases are coordinated) Group 2 plays the role of the staff from a nearby district/supporting Hospital B Group 4 plays the role of the on-site ambulance crew deployed from Hospital A	**Instruction:** This collaborative game requires four players with each player playing different roles. These roles are (1) “Paramedic” (2) “Dispatcher” (or ambulance driver), (3) The doctor in tertiary hospital (“Doctor 1”) and (4) The doctor in district hospital (“Doctor 2”) The ultimate goal of the game is to correctly triage and clear all these patient cards from the “Disaster zone” and the emergency departments of “Tertiary Hospital” and “District Hospital” to the definitive wards within 30 min

For Phase 2, we first performed confirmatory factor analysis using SmartPLS software version 3.3.3 [14] to ensure that the psychometric instrument that was adapted from Ab. Rahman et al. [13] had good validity. Specifically, convergent validity (using factor loadings and average variance extracted or AVE), discriminant validity and internal consistency reliability analyses (using Cronbach’s alpha and composite reliability) of the items were determined [15]. This validated psychometric instrument has five constructs, i.e., (1) “Perceived Usefulness”, (2) “Perceived Ease of Use”, (3) “Attitude”, (4) “Interaction Engagement” and (5) “Behavioral intention” (see Table 2). The detailed process and results of validating the instrument is described in the Supplementary material.

**Table 2:** List of items and constructs in the psychometric instrument

**Construct: Perceived Usefulness Items (PU**						
		1	2	3	4	5
Code	**Item**	**Totally disagree**			**Totally Agree**	
PU1	Using this strategy system improves my overall learning	1	2	3	4	5
PU2	Using this stategy enhances the achievement of learning objectives of this course	1	2	3	4	5
PU3	Using this strategy enhances the achievement of practical skills of this course	1	2	3	4	5
**Construct: Perceived Ease of Use Items (PEOU)**						
PEOU1	This strategy is flexible enough to adopt	1	2	3	4	5
PEOU2	The instruction is clear and easily understandable	1	2	3	4	5
PEOU3	Learning using this strategy does not require a lot of my mental effort	1	2	3	4	5
PEOU4	Overall, I believe this strategy is easy to use	1	2	3	4	5
**Construct: Attitude (A)**		1	2	3	4	5
A1	Overall, I think learning using this strategy is a good idea	1	2	3	4	5
A2	I like learning using this strategy currently	1	2	3	4	5
A3	I look forward to learning using this strategy in future	1	2	3	4	5
**Construct: Interaction Engagement (IE)**						
IE1	This strategy promotes fun	1	2	3	4	5
IE2	This strategy promotes active participation in small-group	1	2	3	4	5
IE3	This strategy allows learner to help each other to learn	1	2	3	4	5
IE4	Asking the lecturer questions when I did not understand	1	2	3	4	5
IE5	I feel that time passed by quickly during this lesson using Tabletop exercise	1	2	3	4	5
IE6	The interaction promoted working together with my teammates	1	2	3	4	5
**Construct: Behavioral Intention**						
MO1	I feel motivated to learn using this approach	1	2	3	4	5
OV1	Overall, I am satisfied to continue adopting this approach of learning.	1	2	3	4	5

Subsequently, using this newly validated psychometric instrument, we compared the medical students’ perception on the tutorless “SMARTriage” board game with the conventional tabletop exercise specifically on “interaction engagement” and “behavioral intention” using a crossover design. Crossover design is a study design where participants are assigned randomly to a sequence of interventions [16]. For example, in the crossover study of AB/BA design, participants would be randomized to receive either intervention “A” followed by “B” or “B” followed by “A”. The advantage of crossover design is that each participant can serve as his or her own control for comparison.

## Results

### Phase 1: Development of the board game (SMARTriage)

**Fig. 1:**
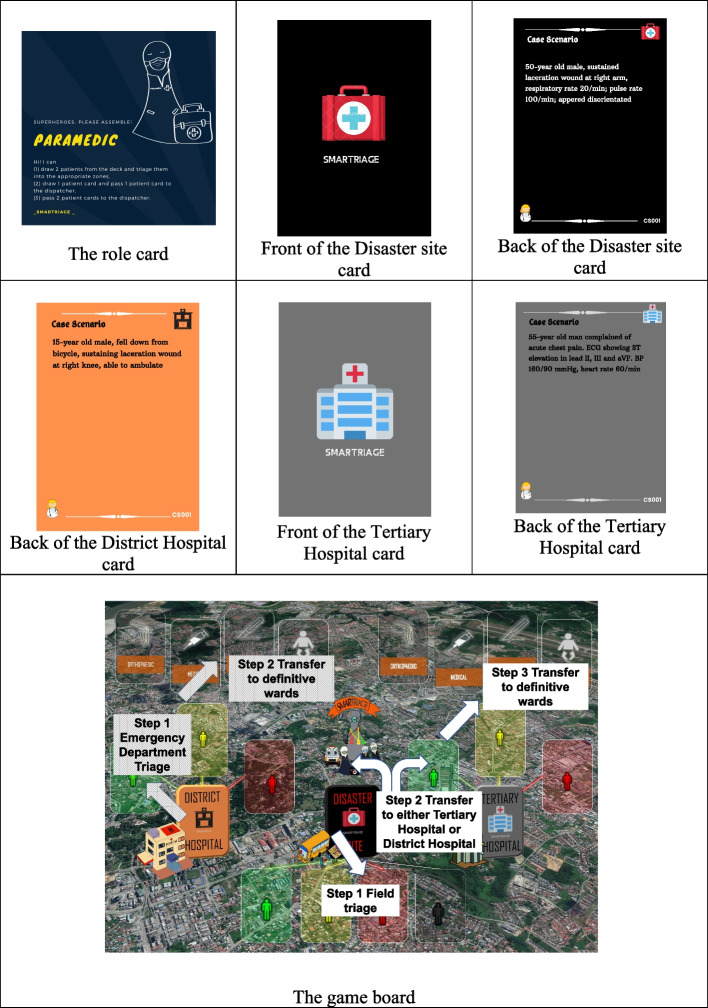
Components of the “SMARTriage” Board Game

Based on the findings from the group discussions, “SMARTriage” board game was designed and developed using the MDA framework by Hunicke et al. (2004). The details of these MDA components are described in Table 3. Examples of the components used in “SMARTriage” board game, i.e., the cards and the game board are illustrated in Fig. 1.

**Table 3:** The Mechanics, Dynamics, Aesthetics and Components of “SMARTriage” Board Game

**Application of MDA Framework in designing the “SMARTriage” board game**
**The Mechanics (or rules) of the game:** 1. This game consists of 4 players, i.e., the “Paramedic”, the “Dispatcher”, “Doctor 1” (who is in charge of the Tertiary or main referral hospital) and “Doctor 2” (who is in charge of the smaller District hospital) 2. Each player is allowed 2 plays for each turn. The “Paramedic” shall start the game first 3. The “Paramedic” can perform field triage for 2 patient cards during his or her turn or triage 1 patient card and initiate transfer by passing another patient card to the “Dispatcher” 4. The main role of the “Dispatcher” is to “transport the patient” from the Disaster site to either the Tertiary or District Hospitals. The “Dispatcher” can also assist the “Paramedic” in field triaging. Hence, the “Dispatcher” can either triage one patient card and “transfer” another patient card or “transfer” 2 patient cards during his or her turn. The “Dispatcher”, however, cannot perform 2 triaging 5. For “Doctor 1” and “Doctor 2”, their jobs are to correctly transfer the patient cards in their respective emergency department to the definitive wards (subject to the handicaps imposed in the game). To know the type of handicaps imposed, roll the dice given and refer the number shown on the dice to the corresponding number on the handicap card 6. “Doctor 1” and “Doctor 2” have the rights to refuse to accept the patient cards from the “Dispatcher” if there is no available bed in their emergency departments 7. For any triaging or transfer that is done wrongly, the card shall return to the bottom of the card deck. This same card can be played again during the subsequent turns. A wrong triage is considered as one play 8. The players check the accuracy of their responses from the answer sheet given
**The Dynamics of the game:**
**The immersion element:** As the ultimate goal of the game is to appropriately triage and clear all 8 patient cards from the disaster site and 4 patient cards in each of the 2 emergency departments of Tertiary hospital and District hospitals within the stipulated 20 min, the players must cooperate and race against time in order to win, thus keeping them immersed in the game
**The challenge element:** To make the game challenging, in every game, both the Tertiary hospital and the District hospital are “handicapped”. For example, if the rolled dice shows ‘2’, the handicap for District Hospital is that “Part of your Red zone is contaminated with a highly infectious micro-organism and has to be closed down. Your Red Zone capacity in Emergency Department is now reduced from 2 to 1 bed.”
**The competence element:** Player must have sufficient prior knowledge on common medical, surgical, obstetrics, pediatric emergencies, etc. to play this game. The checking for the correct answers after the player makes a move provides immediate feedback to enhance learning
**The fun element:** A variety of emergency cases (medical, surgical, obstetrics, pediatric emergencies, etc.) are given in the game to instill the element of fun and surprises
**The social interactions element:** As this is not a competitive game but a collaborative game, the players must work together to navigate through various limitations and challenges to order to win
**The Aesthetics of the game:** The elements of (1) fellowship (how the different players must collaborate and work together) and (2) challenge (how the different players must beat the time in the process of correctly triaging and transferring all patients) are embedded in the game
**Game components:** Game board, 3 sets of cards (one in the “Disaster site”, one in the “Tertiary hospital” and another one in the “District hospital”); dice; stopwatch and colored chips

### Phase 2: Comparison of “SMARTriage” with Conventional Tabletop Exercise

Using Wilcoxon signed rank test, it was that found that overall, tabletop exercise was still rated significantly higher in terms of perceived usefulness, perceived ease of use and behavioral intention compared to tutorless “SMARTriage” board game (refer Table 4). However, in terms of attitude and interaction engagement, there was no significant difference between these two learning methods for most of the items. In particularly, there were no significant difference in 2 out of the 3 items in attitude construct and 5 out of 6 items in interaction engagement construct suggesting that board game was non-inferior in terms of generating interaction engagement among students.

**Table 4:** Comparison of Perception of Tabletop Exercise with Tutorless “SMARTriage” Board Game

		**Median score**		**z-statistics**	***p*-value**
**Factor**	**Item**	**Tabletop Exercise**	**Tutorless “SMARTriage”** **Board Game**		
Perceived Usefulness (PU)	PU1	5.00	4.00	-2.333	0.02
	PU2	5.00	4.00	-2.656	0.01
	PU3	5.00	4.00	-3.225	< 0.001
Perceived Ease of Use (PEOU)	PEOU1	5.00	4.00	-4.520	< 0.001
	PEOU2	4.00	4.00	-5.145	< 0.001
	PEOU3	4.00	3.00	-0.474	0.64
	PEOU4	4.00	4.00	-5.059	< 0.001
Attitude (A)	A1	5.00	5.00	-1.758	0.08
	A2	5.00	5.00	-2.066	0.04
	A3	5.00	5.00	-1.747	0.08
Interaction Engagement (IE)	IE1	5.00	5.00	-0.200	0.84
	IE2	5.00	5.00	-0.671	0.50
	IE3	5.00	5.00	-0.676	0.50
	IE4	5.00	4.00	-4.147	< 0.001
	IE5	5.00	5.00	-0.538	0.59
	IE6	5.00	5.00	-0.144	0.88
Behavioral Intention	MO1	5.00	5.00	-2.294	0.02
	OV1	5.00	5.00	-2.579	0.01

## Discussion

Overall, our findings suggest that students prefer tabletop exercise compared the tutorless “SMARTriage” board game in disaster response training. Specifically, the students rated “perceived usefulness”, “perceived ease of use” and “behavioral intention” significantly higher in tabletop exercise compared to that in the board game. However, a caveat in deciphering the meaning of our findings is the fact that “SMARTriage” board game was intended to be a tutorless, self-contained learning method. While it is true that the proliferation of gamification in teaching and learning has further pivoted the shift from a teacher-centric to a student-centric environment, this study suggests that the presence of tutor is still important in disaster response training. This is consistent with a review by Molin [17] where the author explained how the roles of tutors had often been sidelined or ignored in gamified learning when in fact, tutors play a crucial role to facilitate learning. In this context, the tutor can promote deep and rich collaborative learning rather than simply being an agency of passive knowledge transmission.

Finn and Zimmer [18] highlight on the positive impact of interaction engagement on learning outcomes, suggesting that it leads to improved learning, increased motivation, and better academic performance. Similarly, according to a systematic review by Vlachopoulos and Makri [19], game-based learning approaches confer significant benefits beyond mere cognitive knowledge acquisition. Their findings show that game-based learning not only increases interaction engagement and student motivation, but also promotes the development of critical soft skills such as teamwork, collaboration, organizational skills, adaptability, leadership, as well as a greater ability to resolve conflicts. Consistent with these findings, the rating for most items in the “interaction engagement” construct in “SMARTriage” board game arm were similar with that in the tabletop exercise arm. This suggests that the tutorless “SMARTriage” board game can be as good as tabletop exercise in fostering deep engagement. Previous studies had also shown that gamified learning can be a fun and feasible alternative to foster deep discussion [20, 21] to complement conventional teachings [22, 23].

Few important limitations of this study deserve mentioning. Inherent to the objective of “SMARTriage” to be a tutorless learning activity, the tutor was not present throughout the entire session (unlike in Tabletop exercise). Tutor availability might have confounded the results of the study. To ensure a fairer comparison between these two interventions, future studies could be repeated by controlling for the presence of tutor in both study arms. Secondly, in order to ensure that a game is crisp enough to be completed within a stipulated time bounded by the rules and regulations of the game (“mechanics”), the game has to be necessarily reductionistic. This, however, is unlikely to reflect the complexity of managing disaster in real world. For example, in a real disaster, the physiological status of the patients is dynamic. The patient could deteriorate due to a variety of insults including airway compromises, respiratory fatigue or hemorrhagic shock. This kind of physiological dynamism, however, could not be simulated in a board game; hence, reduces its authenticity. Thirdly, in ideal crossover study design, adequate washout period is recommended until the effect from the first intervention subsides before embarking on the second intervention. In our teaching session, however, due to time restriction, washout period was not possible to be imposed.

Finally, although it was initially mentioned that tabletop exercises may not be suitable in a pandemic as this would require the gathering of the entire group of 40 over students, board game is still not an ideal solution as it would require the gathering of smaller groups of 4 students (although small groups of 4 students would be easier to contain and control). In this regard, a future option would include the creation of a digital or online version of “SMARTriage” board game. This could enable remote gamified learning and exclude any form of physical contact in the event of a pandemic.

## Conclusion

Although a clear preference for tutorless board game was not demonstrated in this study, this study suggests that board game was not inferior to tabletop exercise in fostering interaction engagement. Hence, board game such as the “SMARTriage” could potentially be used as an adjunct for teaching and learning activities.

## Supplementary Information

**Additional file 1.** Validation of the psychometric instrument used in the board game.

## Data Availability

The datasets generated and/or analyzed during this study are not publicly available in order to protect the privacy of our medical students as participants of the study but are available from the corresponding author on reasonable request.
