# Supplementary material for: Tutorless board game as an alternative to tabletop exercise for disaster response training: perception of interaction engagement and behavioral intention
Source: BMC Med Educ. 2023 Jun 12;23:432. doi: 10.1186/s12909-023-04356-4 (PMC10262424; doi:10.1186/s12909-023-04356-4)
Supplement: Supplementary file 1 — Additional file 1. Validation of the psychometric instrument used in the board game. [file 12909_2023_4356_MOESM1_ESM.docx]

**Validation of the Psychometric Instrument used in SMARTriage**

Confirmatory factor analysis using measurement modelling was performed to validate the psychometric instrument developed. Specifically, the convergent validity, discriminant validity and internal consistency reliability analyses of the items were determined. To measure convergent validity, factor loadings and average variance extracted (AVE) were used. Convergent validity refers to the degree to which the items are measuring the same construct. Based on Hair et al (2010), the cut-off values of factor loadings >0.7 and AVE >0.5 are considered as having good convergent validity. To measure discriminant validity, the Fornell and Larcker (1981) criterion was applied. Fornell and Larcker criterion (1981) is fulfilled when the degree to which an item loads on its own construct or factor (as measured using the square root of its AVE value) is higher than its correlation with other constructs or factors (as measured using the square of correlation values).

Internal consistency reliability was assessed using Cronbach’s alpha and composite reliability. For Cronbach’s alpha coefficients, the cut-off value > 0.7 and composite reliability of >0.6 are considered to be acceptable level of internal consistency (Nunally and Berstein 1994).

**Results**

**Validation of questionnaire using measurement model**

Overall, our participants highly rated their motivation to use board game and their overall satisfaction to adopt this approach of learning as 4.48 out of 5 (with standard deviation, SD +/-0.64) and 4.52 out of 5 (SD +/- 0.58) respectively.

Based on the results from the measurement model analysis, we found that all items in our instrument had factor loadings of >0.7 except for PEOU3 and IE4 but as the AVE values were >0.5, the items were not marked for deletion. Internal consistency reliability of the items was good as evidenced by Cronbach alpha and composite reliability of >0.7. For discriminant validity, the Fornell and Larcker criterion (Table 3), was also good indicating that the items in constructs were discriminant enough. No significant cross loading of items in one construct to another construct was noted.


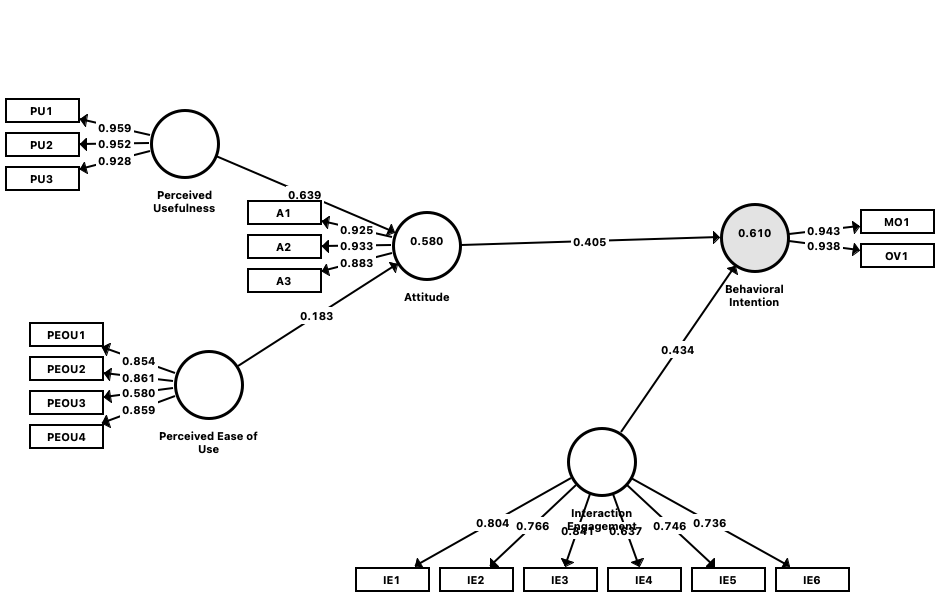


**Table Internal consistency reliability and convergent validity of the constructs**

|  | **Internal Consistency Reliability** | | **Convergent Validity** | | |
| --- | --- | --- | --- | --- | --- |
| **Construct** | **Cronbach’s alpha** | **Composite Reliability** | **Items** | **Factor Loadings** | **Average Variance Extracted (AVE)** |
| **Perceived Usefulness** | 0.94 | 0.96 | PU1 | 0.96 | 0.90 |
|  |  |  | PU2 | 0.95 |  |
|  |  |  | PU3 | 0.93 |  |
|  |  |  |  |  |  |
| **Perceived Ease of Use** | 0.80 | 0.87 | PEOU1 | 0.85 | 0.64 |
|  |  |  | PEOU2 | 0.86 |  |
|  |  |  | PEOU3 | 0.58 |  |
|  |  |  | PEOU4 | 0.86 |  |
|  |  |  |  |  |  |
| **Attitude** | 0.90 | 0.94 | A1 | 0.93 | 0.84 |
|  |  |  | A2 | 0.93 |  |
|  |  |  | A3 | 0.88 |  |
|  |  |  |  |  |  |
| **Interaction Engagement** | 0.85 | 0.89 | IE1 | 0.80 | 0.64 |
|  |  |  | IE2 | 0.77 |  |
|  |  |  | IE3 | 0.84 |  |
|  |  |  | IE4 | 0.64 |  |
|  |  |  | IE5 | 0.75 |  |
|  |  |  | IE6 | 0.74 |  |
|  |  |  |  |  |  |
| **Acceptance** | 0.87 | 0.94 | MO1 | 0.94 | 0.84 |
|  |  |  | OV1 | 0.94 |  |
|  |  |  |  |  |  |

**Figure Discriminant Validity Using Fornell and Larcker criterion of constructs**

|  | **Acceptance** | **Attitude** | **Interaction Engagement** | **Perceived Ease of Use** | **Perceived Usefulness** |
| --- | --- | --- | --- | --- | --- |
| **Acceptance** | **0.94** |  |  |  |  |
| **Attitude** | 0.72 | **0.91** |  |  |  |
| **Interaction Engagement** | 0.73 | 0.73 | **0.76** |  |  |
| **Perceived Ease of Use** | 0.57 | 0.56 | 0.51 | **0.80** |  |
| **Perceived Usefulness** | 0.66 | 0.75 | 0.62 | 0.59 | **0.95** |
